# Diet and Vitiligo: The Story So Far

**DOI:** 10.7759/cureus.28516

**Published:** 2022-08-28

**Authors:** Rajoshee R Dutta, Tanishq Kumar, Nishikant Ingole

**Affiliations:** 1 Medicine, Jawaharlal Nehru Medical College, Datta Meghe Institute of Medical Sciences, Wardha, IND; 2 Pharmacology, Jawaharlal Nehru Medical College, Datta Meghe Institute of Medical Sciences, Wardha, IND

**Keywords:** steroid treatment, herbal supplements, narrowband, puva, dietary supplementation, oxidative stress, pigmentation, melanin, melanocytes, vitiligo

## Abstract

Vitiligo is an acquired skin pigmentation disease with a global burden of 0.5 to 2 percent of the population. Vitiligo therapy frequently poses a difficulty, which has sparked interest in alternative treatment modalities, including multivitamins and herbal supplementation. It has previously been established that nutrition plays a crucial role in developing, amplifying, or rehabilitating an array of human disorders. However, the correlation between diet diversity and immune-mediated skin diseases is still up to interpretation. Several supplements have been studied, including vitamins, minerals, and herbal supplements. Most studies agree that combining vitamin B12, folic acid, and sun exposure is good for inducing repigmentation. Supplementation of zinc and phenylalanine when used in conjunction with topical steroids or UV-B (ultraviolet B) treatment shows therapeutic effects on vitiligo due to their role in the melanin synthesis pathway. Investigations conducted on herbal supplements have revealed that most of them contain antioxidants, which aid in repigmentation. This narrative review's purpose is to discuss nutrition's function in immune-mediated inflammatory skin diseases from the perspective of the most recent and reliable information available.

## Introduction and background

A depigmenting skin pathology, vitiligo leads to hypopigmentation in afflicted regions of the skin by a gradual decrease in melanocytes. Current knowledge of vitiligo pathogenesis has recently advanced and is now categorically recognized as an autoimmune disorder connected with hormonal and hereditary influences in addition to disorders involving metabolism, oxidative stress, and cell disintegration. According to new hypotheses, melanocytorrhagy and poor melanocyte viability are major factors. The hallmark lesion is an amelanotic, non-scaly, chalky-white macule with clear edges [1,2]. The skin commonly affected is that of the face, lips, hands, arms, feet, and genitalia. Additionally, affected areas typically have white hairs. However, the underlying etiopathogenic process of vitiligo is still uncertain [3]. By repairing damaged pigments or removing persisting pigments, vitiligo therapy aims to reduce its severity. Food supplements and a nutrient-dense diet might be seen as essential components of treatment for vitiligo [4]. Although vitiligo is a common condition, it was recently overlooked in dermatology and treated as an ‘orphan illness’ for therapeutic development. Patients frequently claim that doctors are not up to date regarding treatment modalities, and most view vitiligo as a "cosmetic condition” [5]. According to physicians, the focus of future studies should be on novel strategies such as quality of life (QOL) assessments that assess patient satisfaction. 

## Review

Epidemiology

Vitiligo has a reported incidence of 0.5 to 1 percent worldwide [6]. With an estimated 8.8% incidence rate, Gujarat, India, has by far the highest incidence worldwide [7]. Men and women both suffer from the condition [8,9], although women have been found more likely to seek medical assistance. Young women (up to 30 years of age) have a much higher prevalence of vitiligo than young males [8,10]. Women peak by early adolescence, whereas males reach their peak by 45-60 [5].

Types of therapy

One of the most challenging dermatological concerns is currently treating vitiligo. Nevertheless, recent years have seen the development of safe and efficient therapies. Therapies that may slow the condition’s progression, transform depigmented patches, and promote repigmentation include phototherapy, systemic and topical immunosuppressive agents, and surgical procedures [11,12]. The type of vitiligo (segmental or non-segmental), severity, distribution, frequency, age of the patient, type of skin, and willingness to be consistent with therapy are the factors influencing the effectiveness of the treatment. Lips, hands, and feet are likely more resistant to treatment, whereas the head, neck, face, abdominal regions, arms, and legs recover favorably [12]. Repigmentation first develops either at the edges of the lesions or in a specific type of pattern known as “perifollicular." The treatment’s efficacy must be evaluated after at least 2-3 months. The most popular kind of treatment for vitiligo involves UV radiation and, when coupled with other therapies, has been linked to better results [13].

Vitamin B12 and folic acid

Vitamin B12 (also known as cobalamin) constitutes one of the nine water-soluble vitamins and one of the eight vitamin B types [14]. It is one of the most common deficiencies and, if left untreated, might result in blood and nerve disorders [15]. A non-vegetarian diet, including meat, eggs, and dairy products, is a good source of Vitamin B12. The normal B12 consumption is 2.4 μg per day. Only fifty to sixty percent is absorbed [16,17]. Vitamin B12 has been shown to be useful for repigmentation in patients suffering from vitiligo. Folic acid (or vitamin B9) has been proven to be significant for treating vitiligo. It needs to be included in the diet as the body cannot synthesize it. According to an original study conducted in the Birmingham Medical Center, the University of Alabama, 15 patients diagnosed with vitiligo were reported to have low levels of Vitamin B12 and B9. After administering eight of these patients with vitamin B12 and B9 for three years, repigmentation was observed [14]. More research is needed to identify the correct dosage of Vitamin B12 and B9 and the duration for which the skin should be exposed to the sun [18].

Vitamin C

Vitamin C constitutes one of the water-soluble vitamins. Majorly present in citrus fruits like lemon, kiwi, oranges, and green leafy vegetables. Vitamin C should be a part of a balanced diet. It has been indicated that vitamin C has antioxidant action and immunomodulatory characteristics [19,20]. Vitamin C is not used and is contraindicated in treating vitiligo as it disrupts the melanin production pathways [21].

Vitamin D

Vitamin D is a fat-soluble vitamin that absorbs substances like calcium and magnesium. Vitamin D acts on the skin receptors and disrupts the growth and development of melanocytes and keratinocytes [22,23]. 25-hydroxyvitamin D₃ (calcifediol) acts on dihydroxy vitamin D3 receptors on the melanocytes to initiate melanin secretion [24]. According to research, vitamin D levels impact the immune system as the immune system has enzymes/metabolites that can metabolize vitamin D, indicating that the immune system is also contributing to converting inactive forms of vitamin to active forms of calcitriol. This establishes a relationship between the normal functioning of the body’s immune system and circulating vitamin D levels. Any impairment in vitamin D levels would result in disruption of immune system physiology. It can be assumed that dysregulation of the immune system might increase the chances of developing autoimmune diseases. Therefore, if the proper dosage of vitamin D is administered in patients showing vitamin D insufficiency, the outcome of treatment for autoimmune disorders can significantly increase the chances of favoring the patient [25]. Still, insufficient medical evidence indicates that low vitamin D could result in vitiligo. Due to this relation to the immune system, it is highly recommended to include it in the therapy for treating vitiligo. Several studies have been conducted to understand the effect of vitamin D in vitiligo patients. According to a pilot study by Finamor et al., which comprised 16 patients, 35000 IU (international unit) of vitamin D3 was regularly administered every day for six months. Out of 16, 14 patients showed 25% to 75% repigmentation, concluding that supplementation of vitamin D could decrease disease progression [26].

Zinc

More than three thousand proteins, such as hormones, enzymes, and nuclear factors, require zinc as a cofactor for their normal functioning. Superoxide dismutase, a skin antioxidant, uses zinc as an enzyme cofactor [27]. Zinc also controls gene expression. Zinc may also inhibit melanocyte destruction since apoptotic caspases are activated when intracellular zinc concentrations drop [28]. Combined with topical steroids, zinc has been proven to be a marginal advantage in managing vitiligo. Nonetheless, this needs additional investigation. However, treatment-related gastrointestinal adverse effects are a factor that limits zinc supplementation [28]. In an experiment by Yaghoobi et al., 13.3% of zinc-taking participants reported gastric discomfort [29]. Table 1 outlines the properties and impact of the supplements mentioned above on managing vitiligo.

**Table 1:** Summary of Vitamin and Mineral Supplementation in Vitiligo DNA: Deoxyribonucleic acid

Supplement	Properties	Impact on Management of Vitiligo
Vitamin B12 and Folic Acid	DNA synthesis, repair, and methylation	Repigmentation induced with supplementation along with sun exposure, complete repigmentation on following complete therapy
Vitamin C	Antioxidant and immunomodulatory function	Contraindicated as it causes disturbances in the melanin synthesis pathway
Vitamin D	Immune system function	Decreases disease progression when supplemented with standard therapy
Zinc	Cofactor for normal functioning of hormones and enzymes inhibits melanocyte destruction	Provides marginal advantage when combined with topical steroids

Ginkgo biloba

An ancient Chinese plant, *Ginkgo biloba* (GB) has recently acquired considerable attention for its contribution to the treatment of a number of ailments, particularly vitiligo, dementia, macular degeneration, anxiety, and cardiovascular disease [30]. A decrease in cyclooxygenase activity and Tumour Necrosis Factor alpha's role (TNF-a) in inducing the production of interleukin-8 and vascular endothelial growth factor (VEGF) are hypothesized to be the mechanisms of anti-inflammatory effects shown by GB [31]. These qualities shown by ginkgo have been claimed as therapeutic due to the pivotal role of oxidative stress in the pathogenesis of vitiligo. Furthermore, as emotional anxiety was found to aggravate vitiligo, ginkgo’s anxiolytic qualities could slow down the spread of the condition. The majority of individuals consume GB without experiencing any negative side effects, however mild gastrointestinal disturbance is the most frequent side effect. Ginkgo is a viable alternative medicine that has been found to slow the advancement of the illness and enhance repigmentation, according to the findings of two trials.

Polypodium leucotomos

A species of fern called *Polypodium*​​​​​​ *leucotomos* (PL) has been investigated for its significance in the treatment of a number of skin problems, particularly vitiligo, psoriasis, atopic dermatitis, and in preventing UV-induced skin damage. Investigations have been done on the anti-inflammatory, antioxidant, photoprotective, and immunomodulatory properties of PL. When used with phototherapy, ingesting PL is used to boost the efficacy of narrowband UV-B in treating vitiligo [32,33]. It was further established that combining PL with PUVA (psoralen plus ultraviolet-A radiation) treatment results in an increased re-pigmentation. More participants who got >50% re-pigmentation were within the group undergoing PUVA along with PL than the group undergoing PUVA with placebo. All subjects saw the successful treatment of their condition following Anopsos therapy for five months, which is a hydrosoluble lipid derivative of PL [34].

Khellin

Khellin is a crystalline extract from the plant *Ammi visnaga *and it has been utilized in traditional medicine throughout the Mediterranean. Orally administered activated khellin is being studied as a promoter of melanogenesis and proliferation of cultured normal human melanocytes and Mel-1 melanoma cells. These have a possible role in photosensitizing vitiligo treatment when paired with UV therapy. In comparison to no treatment, the combination of 4 percent preparation of topical khellin with monochromatic excimer laser (MEL) treatment at 308 nm, effectively reduced depigmented lesions [35]. Although no discernible difference has been noted in the performance of phototherapy alone and phototherapy with topical khellin, no support substantiates the claimed advantages of topical khellin [36].

Gluten

Celiac disease (referred to as CD) is an autoimmune intestinal infection characterized by individuals who have an adverse reaction to gluten. Damage to the intestinal mucosa, mostly in the form of diarrhea, abdominal discomfort, and other gastrointestinal symptoms, can result from the condition. According to several studies [37,38], people with CD had an increased prevalence of vitiligo than those without CD. Patients who are seropositive for CD immune cells and have autoimmune skin diseases including psoriasis, dermatitis hepatitis, and vitiligo have reportedly experienced fewer symptoms after switching to a gluten-free diet (commonly referred to as GFD) [39-41]. Such type of knowledge is crucial for treating vitiligo patients because the intestinal symptoms are typically vague and frequently disregarded by medical professionals and patients. Additionally, people with vitiligo may benefit from CD screening and CD patients with an early diagnosis of vitiligo may benefit from GFD because it may help both illnesses. To further support these observations, large-scale, long-term follow-up investigations are necessary.

Phenylalanine

The amino acid phenylalanine (Phe) is hypothesized to operate as a possible cure for vitiligo due to its crucial role in the regulation of catecholamine, antibody synthesis, and most importantly, melanin formation. These form the basis of the autoimmune and neurological pathophysiology of vitiligo. Phenylalanine is hydroxylated to tyrosine, which is then used in the process of melanogenesis. Phenylalanine and tyrosine are also closely involved in the production of catecholamines. According to the neural hypothesis, the etiopathogenesis of vitiligo was associated with catecholamines released by autonomic nerve terminals, either directly or indirectly [42]. Phenylalanine or metabolite levels that disrupt catecholamine production may impact vitiligo onset or advancement. Each participant participated as their own control in a clinical investigation that investigated phenylalanine’s impact on vitiligo. After four months of UV-A treatment, the subjects received oral phenylalanine (50 mg/kg) twice a week for the first four months. When the treatments were administered separately, no improvement was detected. Upon administering phenylalanine along with UV-A irradiation, 94.7 percent of individuals exhibited follicular re-pigmentation and 26.3 percent exhibited dense repigmentation [43].

Phyllanthus embelica

*Phyllanthus embelica* Linn., widely recognized as ‘amla fruit’ or Indian Gooseberry is extensively spread in China, India, Indonesia, and Thailand’s tropical and subtropical areas. Research has indicated that *P. emblica* has a strong antioxidant capacity owing to its high polyphenolic component and vitamin C content. *P. embelica* fruit was studied further in 130 subjects in association with carotenoids and vitamin E, which are commonly utilized in vitiligo treatments. In the research, 50 % of participants only got traditional therapies including phototherapy and topical medications. The second section of people received traditional therapy which included combining dietary antioxidants, vitamin E, and carotenoids thrice daily and treatment with topical agents or phototherapy. According to these investigations, a higher percentage of patients in the antioxidant-supplemented group saw minor re-pigmentation in the head, neck, and truck region after six months. Antioxidants were not used in the group that had more erythema, more vitiligous patches, more inflammation, and faster vitiligous zone expansion [44]. 

Piperine

In vitro studies have revealed that piperine, the main alkaloid in black pepper, stimulates melanocyte replication and causes the development of melanocytic dendrites. According to many studies, when UV exposure is present, piperine is recommended. Research has shown the impact of UV light on melanocytes is stimulated by piperin. Piperine only enhanced melanocyte proliferation and dendritic production in melan-a (mouse cell line) when it was not combined with UV-A. Mice given both piperine and UV radiation (UVR) experienced more pronounced pigmentation than mice given either treatment alone. Studies have pointed out that in order to prevent photoisomerization of piperine, UVR and piperine should be used in distinct phases while treating vitiligo [45-47].

Nigella sativa

*Nigella sativa* is a perennial species of plant yielding black cumin, the oil isolates of which are often used to treat a range of illnesses, especially dermatological conditions. Thymoquinone, a primary ingredient of *Nigella sativa* is being carefully researched as a key element possessing a variety of benefits, particularly for its anticancer, immunomodulating, and anti-inflammatory reactions [48-50]. Topical administration with *Nigella sativa* oil has been demonstrated to considerably enhance the Vitiligo Area Scoring Index score within four months [51]. This might be a secure and efficient supplement for conventional vitiligo therapy. 

Punica granatum

One of the first fruit trees that have been planted is the pomegranate (*Punica granatum* Linn.) and it is high in polyphenolic chemicals and tannins. Thus, three to six glasses of commercially accessible pomegranate juice per day could be sufficient to provide antioxidant benefits [52].

Green tea

The polyphenolic molecules known as catechins, which are part of the chemical class of flavonoids, are responsible for green tea's antioxidant properties. Epigallocatechin-3-gallate (EGCG) is by far the most common and therapeutically significant constituent of green tea. It has substantial antioxidant activity as a ROS/RNS (reactive oxygen species/reactive nitrogen species) scavenger with regard to providing potent anti-inflammatory characteristics which can modulate the T-cell-mediated immunological response [53]. Studies demonstrated in two in vitro investigations that EGCG has a potent antioxidant impact on primary human melanocytes. In fact, EGCG reduced ROS production, regenerated impaired mitochondrial function, and lowered apoptosis influenced by hydrogen peroxide. In addition to this, EGCG also controlled oxidative stress-triggered pathways in melanocytes exposed to this stress. Experimental investigations on mice showed depigmentation caused by monobenzone [54]. Studies demonstrate the immune-modulating and oxidative stress-attenuating properties of 2, 5, and 10% EGCG cream. There have not been any trials conducted on how EGCG affects humans so far. Additionally, it was recommended to consume 5 to 16 cups of tea daily to achieve its antioxidant potential. The sole option appears to be EGCG extract supplementation rather than tea infusions [55].

Curcumin

The main naturally occurring lipophilic polyphenol present in the rhizome of *Curcuma longa* (turmeric) and other Curcuma species is curcumin, known as diferuloylmethane. Numerous studies revealed that curcumin shows strong and complex antioxidant activity which enables it to influence the antioxidant system both directly and indirectly as well as inhibiting the generation of ROS and its intracellular sources. One in vivo investigation revealed that when narrowband UV-B (NB-UVB) and tetrahydro-curcuminoid were combined topically on vitiligo patients' skin, the rate of skin repigmentation was marginally greater than when NB-UVB was used alone [56]. Table 2 outlines the properties and impact of the supplements mentioned above on managing vitiligo.

**Table 2:** Summary of Herbal Supplementation in Vitiligo TNF-a: Tumor Necrosis Factor-alpha; PUVA: Psoralen Plus Ultraviolet-A Radiation; UV: Ultraviolet; EGCG: Epigallocatechin gallate; ROS: Reactive oxygen species; NB-UVB: Narrowband Ultraviolet-B

Supplement	Properties	Impact on Management of Vitiligo
Ginkgo biloba	Decrease in cyclooxygenase activity and TNF-a's role in inducing the production of Interleukin-8 and vascular endothelial growth factor (VEGF) are hypothesized to be the mechanisms of anti-inflammatory effects	Enhances repigmentation however mild gastrointestinal disturbances have been noted.
Polypodium leucotomos	Anti-inflammatory, antioxidant, photoprotective, and immunomodulatory properties	Combining *Polypodium leucotomos *with PUVA leads to increased repigmentation
Khellin	Orally administered Khellin plays a role in melanogenesis by stimulating melanocytes	When paired with UV therapy, contributes to a decrease in depigmented lesions
Gluten	May cause inflammation which causes autoimmunity towards melanocytes	Patients with vitiligo may benefit from a gluten-free diet
Phenylalanine	Phenylalanine is hydroxylated to tyrosine which is utilized in melanogenesis. Phenylalanine also causes catecholamine production wherein low catecholamine levels are linked to the onset of vitiligo	Oral phenylalanine supplemented with UVA radiation contributes to increased repigmentation
Phyllanthus embelica	Strong antioxidant property due to high polyphenolic and Vitamin C content	When combined with traditional therapy and appropriate dietary supplementation, contributed to minor repigmentation.
Piperine	Stimulates melanocyte replication and causes the development of melanocytic dendrites	Contributes to patchy repigmentation when combined with UV therapy, but can cause repigmentation when applied without UV therapy
Nigella sativa	Anticancer, immunomodulating, and anti-inflammatory properties	Topical administration considerably increases repigmentation
Punica granatum	High polyphenolic content	3-5 glasses per day provide antioxidant benefits
Green tea	EGCG has substantial antioxidant activity and provides potent anti-inflammatory characteristics which can modulate the T-cell-mediated immunological response. EGCG also regulates mitochondria function	Shows considerable repigmentation when experimented on mice
Curcumin	Exhibits strong antioxidant activity and inhibits ROS production	Topical tetrahydro curcuminoid combined with NB-UVB leads to an increased rate of repigmentation than when NB-UVB is used alone

## Conclusions

Vitiligo is a widespread multifactorial skin condition with complicated pathophysiology. The cause and pathogenesis of vitiligo remain unknown, despite recent significant advancements in our understanding of the condition. In order to find novel treatment targets and medications that could arrest the evolution of the disease or possibly cure vitiligo, it is critical to understand the biological mediators and molecular mechanisms that result in metabolic abnormalities, melanocyte degeneration, and autoimmunity. Numerous alternative treatments, particularly herbal products and vitamin supplements, have been researched to support conventional therapy approaches for vitiligo. Even though several studies have demonstrated benefits linked to these complementary therapies, more extensive and carefully monitored trials are necessary to establish their position in the hierarchy of therapeutic approaches firmly. The most effective treatments included oral *Polypodium leucotomos* with phototherapy, oral ginkgo as monotherapy, and oral phenylalanine as an adjuvant therapy with UV-A therapy.
